# COVID-19 vaccine attitudes and news consumption patterns among pregnant and postpartum individuals in an urban setting

**DOI:** 10.1515/crpm-2025-0005

**Published:** 2025-12-03

**Authors:** Ivana Nikodijevic, Kareena Sagar, Angelica Fiuza, Tara Krishna, Ayana King, Kylie Getz, Damali Campbell

**Affiliations:** 12286 New Jersey Medical School, Newark, NJ, USA; Department of Biostatistics & Epidemiology, Rutgers School of Public Health Piscataway, Piscataway, NJ, USA; Department of Obstetrics, Gynecology and Reproductive Health, Rutgers Health, New Jersey Medical School, Newark, NJ, USA; Department of Obstetrics and Gynecology, Mount Sinai West, New York, NY, USA

**Keywords:** COVID-19, COVID-19 vaccine, vaccine hesitancy, pregnancy, news consumption, social media

## Abstract

**Objectives:**

Pregnant individuals face increased COVID-19 symptom severity, yet vaccination rates remain low. The study aimed to identify strategies for improving vaccine adherence by examining pregnant individuals’ attitudes toward the COVID-19 vaccine and their news consumption habits.

**Case presentation:**

A total of 58 pregnant and postpartum individuals were surveyed in the Obstetrics & Gynecology clinic at an urban academic medical center in Newark, NJ from June to December 2023. The 88-item survey assessed sociodemographic characteristics, vaccination status, pandemic-related risk perception, resource accessibility, and news sources. Sixty-six percent of participants reported receiving the COVID-19 vaccine. Vaccinated individuals were more likely to agree with pro-vaccine statements. Many respondents, regardless of vaccination status, selected “neither agree nor disagree” for anti-vaccine claims including those related to infertility or miscarriage. Participants who preferred traditional news sources (e.g. television broadcasts) were more likely to support pro-vaccine statements, while social media users expressed greater uncertainty.

**Conclusions:**

Concerns about the safety and efficacy of the COVID-19 vaccine were key in hesitancy among pregnant individuals, with social media contributing to this hesitancy. Our study highlights the need for holistic and effective communication from healthcare providers, evidence-based information across media platforms, and increased vaccine accessibility to improve vaccine adherence.

## Introduction

Pregnancy is a unique time when individuals are especially receptive to health education and motivated to adopt health-seeking behaviors for themselves and their baby. Antenatal care offers an important opportunity for patient-provider discussions, focusing on healthy behaviors and potential complications [1]. Surprisingly, one of the largest COVID-19 vaccination gaps to emerge has been amongst pregnant persons despite the increased risk of severe COVID-19 infection during pregnancy [2], 3]. [1] As of July 2024, the Centers for Disease Control and Prevention (CDC) reported a significantly lower percentage of pregnant persons have received the updated COVID-19 vaccine, with even lower rates among persons of color – 7.8 % for Hispanic individuals and only 5.9 % of Black individuals [4].

This gap in vaccination is of particular concern because pregnant and postpartum patients with a COVID-19 infection have an increased risk of intensive care unit admission, need for mechanical ventilation, preterm delivery, and neonatal intensive care unit admission of their babies [3]. Despite these complications and guidelines endorsing that the COVID vaccines are safe during pregnancy, general hesitancy remains [5], 6].

### News consumption

The widespread use of the internet and media ensures COVID-19 vaccination information is readily available, but it also facilitates the spread of misinformation and conspiracy theories about the vaccine [7], [8], [9]. Social media has been used as a platform to amplify the anti-vaccine movement, pushing theories such as associations with 5G, inserting microchips, and causing severe adverse effects and death [6]. Many studies have shown the use of social media is associated with increased COVID-19 vaccine hesitancy, in part because individuals struggle to discern between true and false content [10], [11], [12]. Specifically in the pregnant and postpartum population, studies in Brazil and Jordan have found that reasons for rejecting the vaccine include news read on the internet and that pregnant women were more likely to receive the vaccine if their main information source was a news channel or a health ministry [13], 14].

### Low-resource communities

The specific population of this study is individuals in the community of Newark, NJ, a highly diverse and a lower-resourced community. Per 2020 census data, 16.3 % of the population is without health care coverage compared to 6.8 % of the total New Jersey state population [15]. 47.4 % of the Newark, NJ population speaks another language other than English at home and the median household income is $49,688 while the state median household income is $96,346 [15]. These factors play a role in how people within this community access and interact with the local health care system. One study found that, in the greater Newark area, being a Spanish speaker with limited English proficiency was suggested to be an independent risk factor for delaying care [16]. In addition to lower socioeconomic status and language barriers, factors that may limit healthcare access also include lack of trust in healthcare systems and misinformation or lack of information about health services [17]. However, there are limited studies as to how pregnant individuals within low-resource communities are particularly impacted by these barriers.

It is important to understand the reason for this vaccine hesitancy to more efficiently counsel and target vaccine adherence in this population. Prior surveys in pregnant people have found that vaccine safety is the most cited concern preventing acceptance of the COVID-19 vaccine [18], 19]. In our review of the literature, we did not find any US studies that have examined the effects of news consumption on the COVID-19 vaccine attitudes of pregnant and postpartum people, especially in low-resourced highly diverse populations.

The primary purpose of this study is to identify associations between beliefs, behaviors, and resources among pregnant and recently postpartum people, and their vaccination status against COVID-19. Secondarily we sought to identify potential barriers to vaccination. This will inform the medical community on potential avenues for public education and intervention in order to increase vaccination rates within this vulnerable population. This is an ongoing issue of the need to improve vaccination among pregnant persons and for future public health emergencies.

### Statement of inclusivity

We recognize that all pregnant individuals may not identify as women. We used inclusive language throughout the document but left “pregnant women” when referring to source literature.

## Materials and methods

Pregnant and recently postpartum persons in the OB/GYN clinic of University Hospital, Newark, NJ were approached to complete a survey. The 88-item survey was available in online and paper format, in English and Spanish, and took around 15 min to complete. Surveys were administered to prospective participants both in the clinic waiting room and smaller waiting areas outside the ultrasound suite and patient rooms. Prior to survey completion, a request for volunteer participation and consent form was administered. The survey questions examined participants’ sociodemographic characteristics such as age, race/ethnicity, education level, household income, employment status, insurance type, and religious affiliation. Additionally, the survey assessed vaccination status, impact of the COVID-19 pandemic, pandemic associated risk perception, resource accessibility, and news consumption patterns. Patient information used in the study was encrypted with identification codes used in place of the patient’s name. The initial intention was to have a multi-site study, however other sites had difficulty recruiting staff due to workforce challenges. Data collection at University Hospital spanned from June to December 2023. Due to the nature of the survey questions and the delays in its administration, the survey did not distinguish whether a participant received the COVID-19 vaccine during pregnancy or prior to pregnancy. Ethical approval for this research was granted by the internal review board (IRB) of Rutgers University and University Hospital located in Newark, NJ and is under ID Mod2023001354.

### Data analysis

Statistical analysis was performed using the statistical software package R, version 4.2.1. All categorical measures were summarized using frequencies and percentages and were compared using either the Chi-squared test or Fisher’s exact test. Ordinal variables were compared using a Wilcoxon rank sum test (e.g. disagree, neutral, agree) as there is an order to the rank. Likert plots were generated using the Likert package in R.

Multiple variables were recategorized. The variables assessing agreement were grouped as follows: ‘strongly disagree’ and ‘somewhat disagree’ were combined into ‘disagree’; ‘somewhat agree’ and ‘strongly agree’ were combined into ‘agree’; and ‘neither agree nor disagree’ was kept as its own distinct category. ‘Did not complete secondary/high school’, ‘High School/GED’, ‘Some College’, ‘Professional qualification/Trade School’ were combined into ‘trade or less than bachelor’s’, while ‘bachelor’s degree or similar’ and ‘Masters or Doctoral degree’ were combined into ‘Bachelor’s or higher’. Household income of $50 k-$99 k and >$100 k were combined into >$50 k.

## Results

A total of 68 surveys were administered, of which 62 were completed. Of the completed surveys, 58 respondents answered the question regarding COVID-19 vaccination status and were therefore included in our analysis. Thirty-six percent of participants included in our analysis were Spanish-speaking. Sixty-six percent of respondents reported receiving the COVID-19 vaccine.

### Demographics

Hispanic individuals comprised the largest ethnic group in our sample, representing 50 % of respondents. There was a significant association between identifying as Hispanic and receiving the COVID-19 vaccine (p=0.014). Race was a significant predictor of vaccine acceptance (p<0.001) where fewer Black/African-American respondents received the vaccine compared to White, Hispanic/Latino, and Asian patients. There was a trend towards higher vaccination rates among individuals with a bachelor’s degree or higher compared to those with less education (p=0.072). A similar pattern was observed with income, as individuals reporting annual income greater than $50,000 were more likely to be vaccinated than those earning less (p=0.061) (Table 1). There were no significant differences in age, ethnicity, race, education level, or income across participants’ preferred sources of news.

**Table 1:** Demographic characteristics of 58 pregnant and post-partum patients presenting to the OB-GYN clinic.

	Total n	Not vaccinated n, %	Vaccinated n, %	p-Value
Age, years				0.9
16–19	4	1 (25)	3 (75)	
20–29	32	11 (35)	20 (65)	
30–39	15	6 (40)	9 (60)	
40–49	3	0 (0)	3 (100)	
50–59	1	0 (0)	1 (100)	
Ethnicity				0.014
Hispanic	29	5 (17)	24 (83)	
Non-hispanic	20	10 (50)	10 (50)	
Race^a^				<0.001
Asian	2	0 (0)	2 (100)	
Black	21	14 (67)	7 (33)	
Hispanic	2	0 (0)	2 (100)	
Other	13	2 (15)	11 (85)	
White	14	1 (7.1)	13 (93)	
Education level				0.072
Bachelors or higher	11	1 (9.1)	10 (91)	
Trade or less than bachelors	40	17 (43)	23 (58)	
Unknown	7	2	5	
Household income				0.061
$0 k-$49 k	33	16 (48)	17 (52)	
>$50 k	10	1 (10)	9 (90)	
Unknown	15	3	12	
Religion				0.6
Christianity	23	9 (39)	14 (61)	
None	3	2 (67)	1 (33)	
Unknown	32	9	23	

^a^Self-identified data.

### COVID-19 and vaccination personal and family history

**Figure 1:**
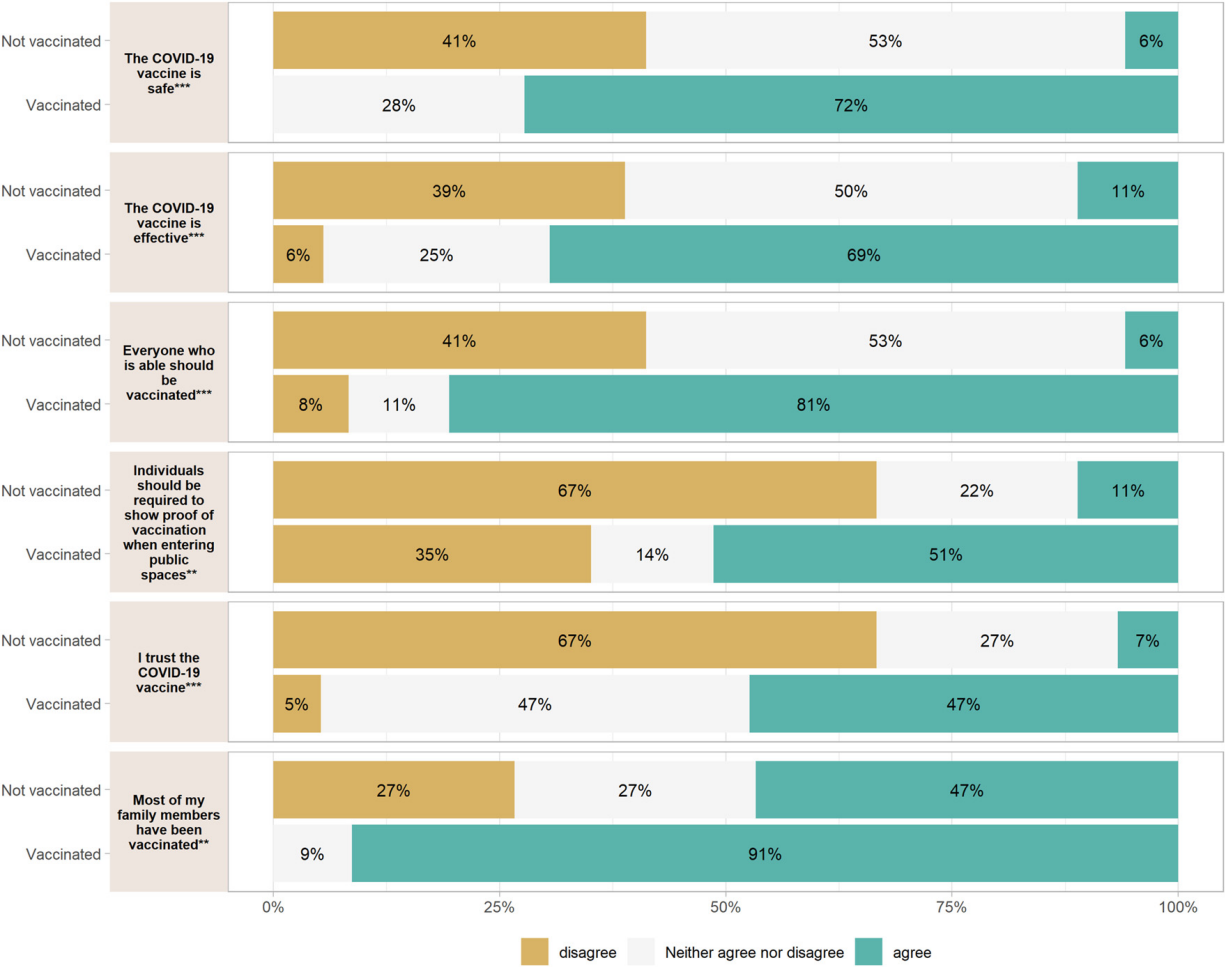
Vaccination status and responses to statements regarding COVID-19 and vaccination personal and family history. *p<0.05, **p<0.01, ***p<0.001.

There were no significant differences in vaccine status among individuals who reported having COVID-19 in the past, or who knew someone that passed away from COVID-19. There were no differences in individuals who reported receiving all their childhood immunizations or reported plans to receive the flu shot. There was a significant difference in responses to the statement “Most of my family members have been vaccinated” (p=0.002) with more vaccinated individuals agreeing with the statement than unvaccinated individuals (Figure 1).

### Personal attitudes and beliefs on COVID-19

100 % of unvaccinated individuals agreed with the statement “people who are sick with a cold should isolate themselves until they are better” (p=0.005). There were no differences in vaccination status among responses to “I have good resources to learn information about COVID-19”. 0 % of individuals disagreed with this statement.

### Personal attitudes and beliefs on vaccination

There was a significant difference among responses to the statement “the COVID-19 vaccine is safe” (p<0.001). Vaccinated individuals were more likely to agree than unvaccinated individuals. Vaccinated individuals were also more likely to agree to the statement “the COVID-19 vaccine is effective” (p<0.001). There was a significant difference in responses to the statement “everyone who is able should be vaccinated” (p<0.001) where more vaccinated individuals agreed than unvaccinated individuals. The statement “Individuals should be required to show proof of vaccination when entering public spaces (such as theaters, restaurants, sporting events.)” also had a significant difference (p=0.004) where more vaccinated individuals agreed than unvaccinated individuals. Similarly, with the statement “I trust the COVID-19 vaccine” (p<0.001) more vaccinated individuals agreed (Figure 1).

### Anti-vaccine statements

**Figure 2:**
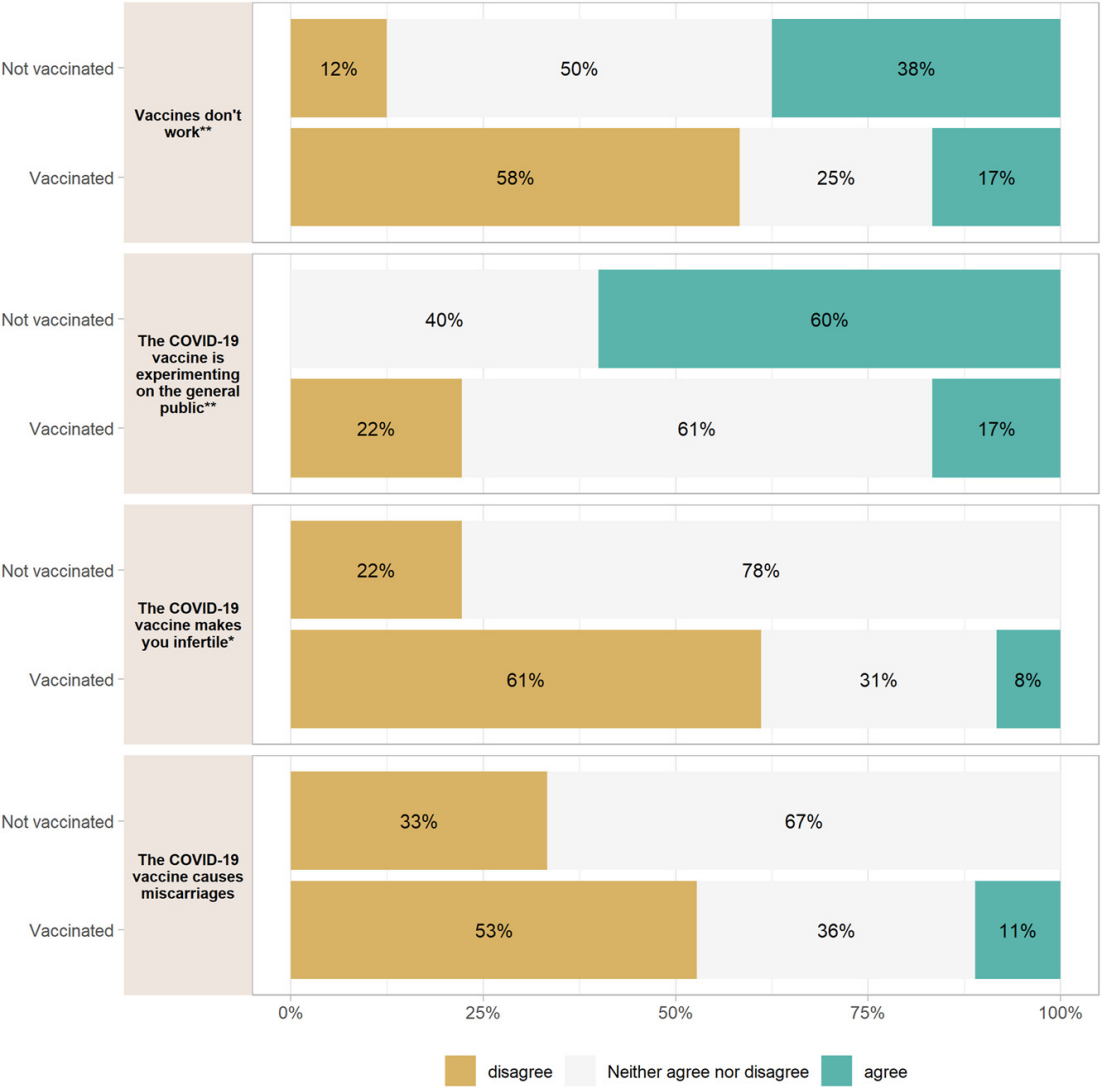
Vaccination status and responses to anti-vaccine statements. *p<0.05, **p<0.01, ***p<0.001.

**Figure 3:**
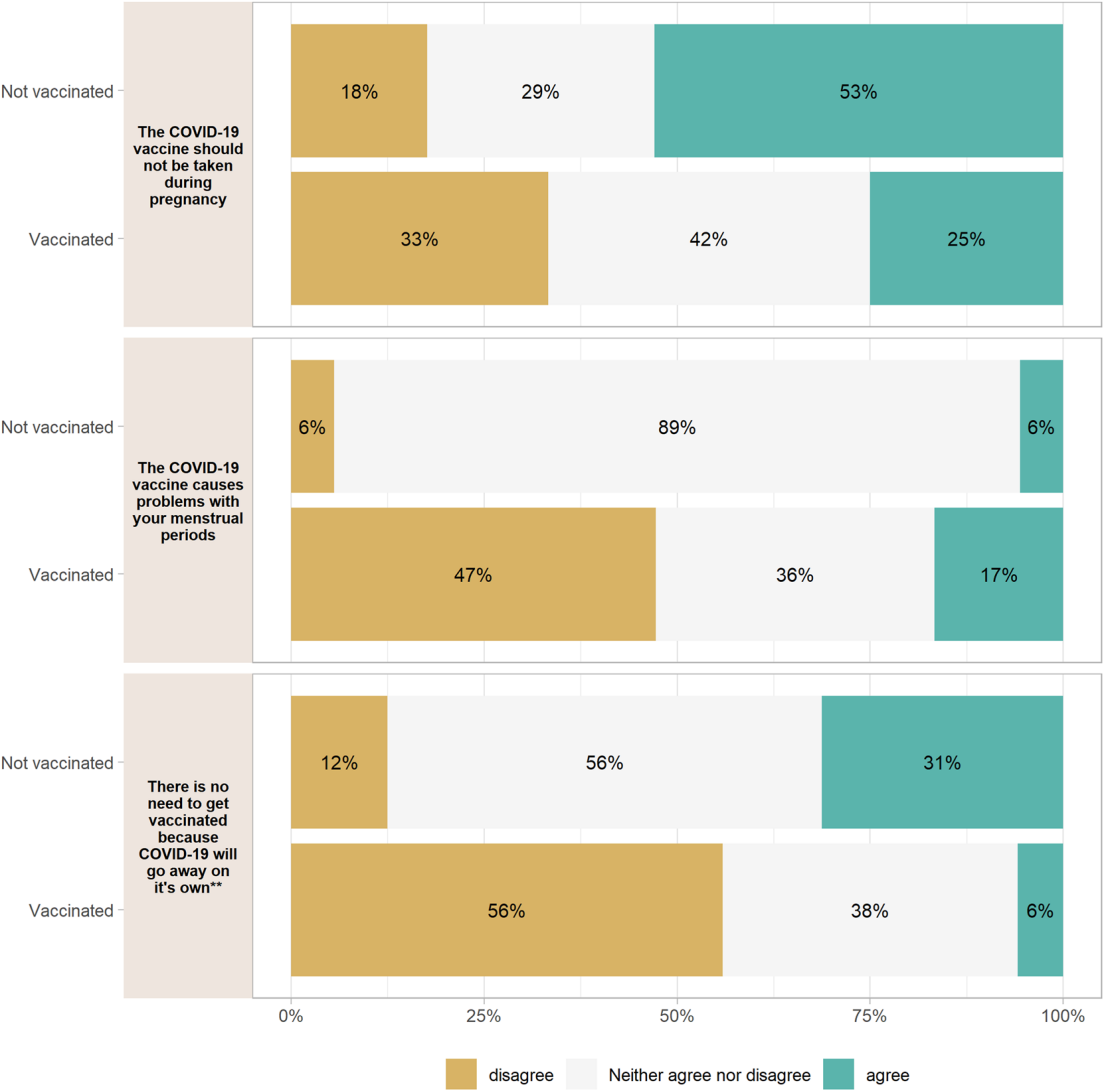
Vaccination status and responses to anti-vaccine statements, with large percentages responding “neither agree nor disagree”. *p<0.05, **p<0.01, ***p<0.001.

There was a significant difference among responses to the statement “Vaccines don’t work” (p=0.004) where more vaccinated individuals disagreed than unvaccinated individuals. There was a difference in responses to the statement “the COVID-19 vaccine is experimenting on the general public” (p=0.005). 100 % of those who disagreed with the statement were vaccinated. The statement “the COVID-19 vaccine makes you infertile” had a significant difference in responses (p=0.031) with more vaccinated individuals disagreeing with the statement. 48 % of respondents responded “neither agree nor disagree” to this statement. 48 % of respondents also responded “neither agree nor disagree” to “the COVID-19 vaccine causes miscarriages” (Figure 2). There was an association the statement “the COVID-19 vaccine should not be taken during pregnancy” (p=0.061), with more vaccinated individuals disagreeing with the statement. 39 % of participants responded “neither agree nor disagree” to this statement. There was an association between vaccination status and disagreement to the statement “the COVID-19 vaccine causes problems with your menstrual period” (p=0.057), with more vaccinated individuals disagreeing. 56 % of participants responded “neither agree nor disagree” to this statement. There was a significant difference in responses to the statement “there is no need to get vaccinated because COVID-19 will go away on its own” (p=0.001) with more vaccinated individuals disagreeing (Figure 3).

### News consumption

**Figure 4:**
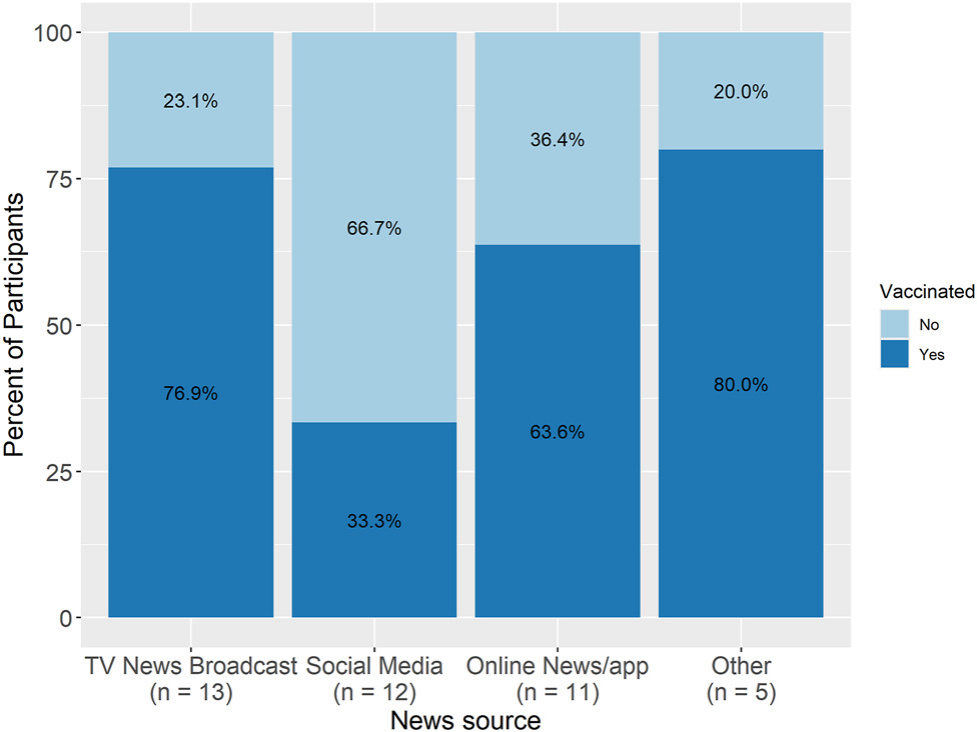
Vaccination status and preferred news source.

Of the participants included in the analysis, 41 provided information on their preferred news source and were included in the news consumption analysis. The #1 preferred source for updated information about healthcare/COVID-19 was TV News Broadcast in 32 % of responders, Online News/App in 27 % of responders, social media in 29 % of responders, and Other in 12 % of responders. Out of those that responded TV News Broadcast, 77 % received the vaccine and 23 % did not. Within Online News/App, 63 % received the vaccine and 36 % did not. Within social media, 33 % received the vaccine and 67 % did not (Figure 4).

There was a significant difference in responses to pro-vaccine statements like “the COVID-19 vaccine is safe” (p=0.030) and “Everyone who is able should be vaccinated” (p=0.006) with more individuals who prefer TV news broadcast agreeing and individuals who prefer social media disagreeing. In response to the statement “Vaccines don’t work”, those who prefer social media were more likely to agree, and TV news broadcast and online news/app were more likely to disagree (p=0.010). Many individuals who prefer social media were more likely to respond “neither agree nor disagree” to statements like “the COVID-19 vaccine causes problems with your menstrual periods” (p=0.020) and “the COVID-19 vaccine makes it harmful to breastfeed” (p=0.006), with more individuals who prefer TV news broadcast and online news/app disagreeing with these statements. Participants who prefer TV news broadcast and social media were both more likely to respond “neither agree nor disagree” to statements like “the COVID-19 vaccine causes autism” (p<0.001), “COVID-19 was created as a biological weapon” (p=0.026), “the COVID-19 vaccine causes miscarriages” (p=0.021), and “COVID-19 is a plot by Big Pharma” (p=0.002), with individuals who prefer online news/app more likely to disagree with these statements (Table 2).

**Table 2:** Preferred source of news and anti-vaccine beliefs of participants.

	TV news broadcast n=13	Online news/app n=11	Social media n=12	p-Value
Vaccines don’t work				0.010
Disagree	6 (46)	5 (50)	0 (0)	
Neither agree nor disagree	4 (31)	5 (50)	5 (42)	
Agree	3 (23)	0 (0)	7 (58)	
The COVID-19 vaccine makes you magnetic due to 5G telecommunication towers				0.5
Disagree	3 (23)	5 (45)	4 (40)	
Neither agree nor disagree	9 (69)	4 (36)	6 (60)	
Agree	1 (7.7)	2 (18)	0 (0)	
The COVID-19 vaccine is experimenting on the general public				0.2
Disagree	1 (13)	3 (27)	0 (0)	
Neither agree nor disagree	4 (50)	5 (45)	4 (40)	
Agree	3 (38)	3 (27)	6 (60)	
I Trust most vaccines, but not the COVID-19 vaccine				0.058
Disagree	2 (25)	4 (36)	0 (0)	
Neither agree nor disagree	2 (25)	6 (55)	9 (82)	
Agree	4 (50)	1 (9.1)	2 (18)	
The COVID-19 vaccine makes you infertile				0.6
Disagree	4 (31)	6 (55)	3 (27)	
Neither agree nor disagree	9 (69)	5 (45)	8 (73)	
Agree	0 (0)	0 (0)	0 (0)	
The COVID-19 vaccine causes miscarriages				0.021
Disagree	3 (23)	8 (73)	1 (8.3)	
Neither agree nor disagree	9 (69)	3 (27)	11 (92)	
Agree	1 (7.7)	0 (0)	0 (0)	
The COVID-19 vaccine causes birth deformities				0.004
Disagree	3 (23)	8 (73)	0 (0)	
Neither agree nor disagree	9 (69)	3 (27)	12 (100)	
Agree	1 (7.7)	0 (0)	0 (0)	
The COVID-19 vaccine causes problems with your menstrual periods				0.020
Disagree	5 (38)	6 (55)	0 (0)	
Neither agree nor disagree	8 (62)	5 (45)	11 (92)	
Agree	0 (0)	0 (0)	1 (8.3)	
The COVID-19 vaccine makes it harmful to breastfeed				0.006
Disagree	4 (33)	7 (64)	1 (8.3)	
Neither agree nor disagree	7 (58)	4 (36)	8 (67)	
Agree	1 (8.3)	0 (0)	3 (25)	
The COVID-19 vaccine causes autism				<0.001
Disagree	2 (15)	8 (73)	0 (0)	
Neither agree nor disagree	9 (69)	3 (27)	10 (91)	
Agree	2 (15)	0 (0)	1 (9.1)	
The COVID-19 vaccine will cause long-term complications				0.078
Disagree	1 (7.7)	5 (45)	0 (0)	
Neither agree nor disagree	9 (69)	5 (45)	10 (83)	
Agree	3 (23)	1 (9.1)	2 (17)	

Percentages are by column, denominators are total of each response group.

There was no significant difference among news consumption habits with statements that discuss the impact of COVID-19 such as “the COVID-19 pandemic has caused millions of deaths worldwide” (p>0.9) and “COVID-19 is a serious illness and health risk” (p=0.8) (Table 3).

**Table 3:** Preferred source of news and COVID-19 pandemic beliefs of participants.

	TV news broadcast n=13	Online news/app n=11	Social media n=12	p-Value
COVID-19 was created as a biological weapon				0.026
Disagree	1 (7.7)	5 (45)	0 (0)	
Neither agree nor disagree	9 (69)	5 (45)	7 (64)	
Agree	3 (23)	1 (9.1)	4 (36)	
COVID-19 is a plot by big pharma				0.002
Disagree	3 (23)	6 (55)	0 (0)	
Neither agree nor disagree	9 (69)	5 (45)	10 (83)	
Agree	1 (7.7)	0 (0)	2 (17)	
The COVID-19 pandemic has caused millions of deaths worldwide				>0.9
Disagree	0 (0)	0 (0)	1 (8.3)	
Neither agree nor disagree	2 (15)	2 (18)	1 (8.3)	
Agree	11 (85)	9 (82)	10 (83)	
COVID-19 is a serious illness and health risk				0.8
Disagree	0 (0)	0 (0)	1 (8.3)	
Neither agree nor disagree	2 (15)	1 (9.1)	1 (9.3)	
Agree	11 (85)	10 (91)	10 (83)	

n (%) - indicates percentage of individuals within each news source. Percentages are by column, denominators are total of each response group.

## Discussion

### News consumption

The #1 preferred news source for updated information on healthcare/COVID-19 was evenly split among respondents between TV news broadcast, online news/app, and social media. Consistent with prior studies, more traditional news sources, such as TV news broadcasts and online news/apps, were more popular among those that received the COVID-19 vaccine, while social media was more popular among those that did not [10], 12].

Participants who identified more traditional news sources as their preferred news source were more likely to agree to pro-vaccine statements such as the COVID-19 vaccine is safe and effective, and “everyone who is able should be vaccinated”. They were also more likely to disagree with anti-vaccine statements such as “vaccines don’t work”. Interestingly, nearly 100 % of participants who preferred social media responded “neither agree nor disagree” to many anti-vaccine statements, including claims that the COVID-19 vaccine causes miscarriages, problems with menstrual periods, autism, long-term complications, makes it harmful to breastfeed, and that COVID-19 is a plot by Big Pharma. The fact that more social media users responded “neither agree nor disagree” than traditional news users indicates social media may be causing more uncertainty and leading participants to be less likely to disagree with conspiracy theories.

Islam et al. analyzed the accuracy of COVID-19-related information on social media and found that 82 % of claims were false [8]. This is compounded by the “algorithms” of social media where viewers primarily see content that aligns with their views, perpetuating the anti-vaccine movement. To counter this, more robust efforts are needed to promote evidence-based content on social media, particularly campaigns tailored to address the concerns of pregnant individuals. While such campaigns have been conducted to date, further research is needed to explore their effectiveness in improving vaccine adherence [20], 21].

Although participants that prefer social media as their news source are less likely to receive the vaccine and more likely to agree to anti-vaccine statements, almost all still agreed to statements that COVID is a dangerous disease and that it has caused millions of deaths worldwide. As a result, it appears that the reasons for their anti-COVID vaccine sentiments are fears over the vaccine itself, rather than the belief that it is unnecessary.

### Vaccine safety and effectiveness

Our findings suggest several ways to improve COVID-19 vaccine adherence in pregnant patients, including education on vaccine safety and effectiveness. Patients in our study who viewed the vaccine as safe and effective were significantly more likely to be vaccinated. This supports prior research showing that perceived safety for the patient and fetus is a key factor in vaccine uptake during pregnancy [22], [23], [24], [25], [26]. Bonilla et al. (2023) found that pregnant patients who accepted the vaccine were more knowledgeable of how vaccines work and the factors increasing risk of symptom severity [26].

The need for more education on vaccine safety in pregnant and postpartum people is also evident in the frequent “neither agree nor disagree” responses to anti-vaccine statements, regardless of vaccination status. This pattern again suggests that participants were likely uncertain about the accuracy of these statements. Such uncertainty toward vaccination during pregnancy raises the importance of preconception counseling, when individuals may be more open to vaccination.

Participants in our study, regardless of vaccination status, generally felt they had access to reliable resources for COVID-19 information despite agreeing with false statements. This highlights that individuals continue to hold misconceptions even when they believe their information sources are credible. These findings emphasize the need for targeted discussions with pregnant patients to examine their information sources and address misinformation. Simple yet impactful measures like offering tip sheets, discussing what to expect after vaccination, and conducting follow-up calls or sending automated text messages can help build trust and alleviate fear within the clinical setting.

### Barriers to vaccination

While the novelty of the vaccine and concerns over its safety and effectiveness play a role in vaccine hesitancy, accessibility may also be a contributing factor. Clinics often lack the COVID-19 vaccine during appointments due to storage issues, unlike more readily available vaccines like the flu shot or Tdap. Improving access to the COVID-19 vaccine, compared to more widely accepted vaccines, along with more focused promotional efforts, is needed.

Our findings suggest higher vaccination rates among individuals with greater education and income, consistent with prior studies showing that patients with lower educational attainment are more likely to delay or refuse vaccination [27]. People with lower educational attainment often hold jobs without paid sick leave or schedule flexibility, limiting access to healthcare during standard hours. Fear of vaccine side effects may also deter them, as missing work is unaffordable. Strategies such as community vaccination sites, evening and weekend appointments, and employer collaborations to promote vaccination or provide onsite services, could address these challenges. Our findings also support existing research suggesting that individuals are more likely to be vaccinated against COVID-19 when their family members are also vaccinated [28]. Therefore, promoting vaccination efforts among non-pregnant individuals may have a positive effect on vaccine uptake within our target population.

### Limitations

There were some limitations in data collection and analysis that may alter the study’s conclusions. One limitation is that data collection, spanning June to December, 2023, occurred about two years after the release of the original COVID-19 vaccine rollout. When respondents answered “Yes” to having received the vaccine, it referred to any point in their lifetime. Thus, our results reflect attitudes toward the vaccine among individuals of reproductive age rather than exclusively pregnant, postpartum, or breastfeeding people. Future studies could address this by asking if respondents received the vaccine specifically during these periods. Despite this limitation, our conclusions remain valuable in establishing that individuals with greater trust in healthcare were more likely to be vaccinated and the role of news sources in vaccine decisions.

Another limitation of the study is the small sample size, which resulted from IRB delays and the use of a single data collection site. Still, our study yielded several significant findings. A larger sample size could have further strengthened these results and potentially revealed additional significant associations.

Additionally, some respondents left a few of the 88 survey questions blank, reducing the sample size and potentially weakening the power of those analyses. Future research can address this by identifying why certain questions were unanswered. Pre-survey focus groups with representatives of the study population could improve question comprehension and quality, reducing these gaps.

## Conclusions

This study identified key factors contributing to vaccine hesitancy among pregnant and postpartum individuals in Newark, NJ. The primary barrier was uncertainty about vaccine safety and efficacy for the patient and fetus. Healthcare providers must clearly communicate the COVID-19 vaccine’s safety during pregnancy and its benefits in mitigating severe COVID-19 risks. Addressing patient concerns can reduce vaccine apprehension in this vulnerable population. Additionally, improving COVID-19 vaccine accessibility to match that of more readily available vaccines is crucial to increasing uptake.

This study revealed that patients in this population receive their news from a variety of sources. Thus, it is important to distribute evidence-based content about the COVID-19 vaccine across all platforms, including TV news, online news/apps, and social media. However, given the link between social media and vaccine hesitancy, trusted organizations and providers must focus on delivering evidence-based information especially through social media to address concerns about the vaccine in this population.
